# A cancer derived mutation in the Retinoblastoma gene with a distinct defect for LXCXE dependent interactions

**DOI:** 10.1186/1475-2867-10-8

**Published:** 2010-03-18

**Authors:** Shauna A Henley, Sarah M Francis, Jordan Demone, Peter Ainsworth, Frederick A Dick

**Affiliations:** 1 London Regional Cancer Program, 790 Commissioners Road East, London, Ontario, N6A 4L6, Canada; 2 Children's Health Research Institute, 800 Commissioners Road East, London, Ontario, N6A 4L6, Canada; 3 Department of Biochemistry, University of Western Ontario, 1151 Richmond Street, London, Ontario, N6A 5B8, Canada; 4 Molecular Diagnostics Laboratory, London Health Sciences Centre, 800 Commissioners Road East, London, Ontario, N6A 4L6, Canada

## Abstract

**Background:**

The interaction between viral oncoproteins such as Simian virus 40 TAg, adenovirus E1A, and human papilloma virus E7, and the retinoblastoma protein (pRB) occurs through a well characterized peptide sequence, LXCXE, on the viral protein and a well conserved groove in the pocket domain of pRB. Cellular proteins, such as histone deacetylases, also use this mechanism to interact with the retinoblastoma protein to repress transcription at cell cycle regulated genes. For these reasons this region of the pRB pocket domain is thought to play a critical role in growth suppression.

**Results:**

In this study, we identify and characterize a tumor derived allele of the retinoblastoma gene (*RB1*) that possesses a discrete defect in its ability to interact with LXCXE motif containing proteins that compromises proliferative control. To assess the frequency of similar mutations in the *RB1 *gene in human cancer, we screened blood and tumor samples for similar alleles. We screened almost 700 samples and did not detect additional mutations, indicating that this class of mutation is rare.

**Conclusions:**

Our work provides proof of principal that alleles encoding distinct, partial loss of function mutations in the retinoblastoma gene that specifically lose LXCXE dependent interactions, are found in human cancer.

## Background

**Figure 1:**
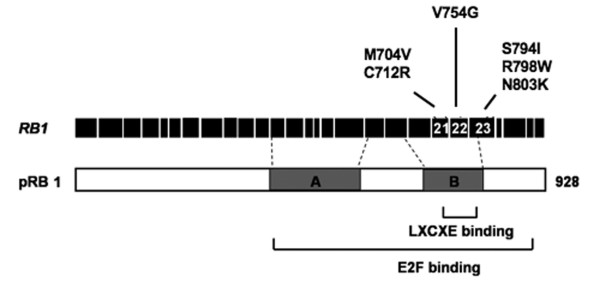
**Schematic diagram of the pRB open reading frame and corresponding exon structure of the *RB1 *gene**. Candidate mutations in exons 21, 22, and 23 that were examined in this study are shown.

The *RB1 *gene was first identified based on its mutation in retinoblastoma [1,2], however, it has since been found to be absent or misregulated in almost all human cancers [3]. The retinoblastoma protein (pRB) is also thought to be an essential target for inactivation in cancer because it is targeted by viral oncogenes during cellular transformation. The TAg protein product of Simian virus 40, E1A from adenovirus, and E7 from human papilloma virus all contain an LXCXE peptide motif that is essential for transforming activity and it is used to contact the pocket domain of pRB [4-6]. A number of cellular proteins, most notably chromatin regulators such as histone deacetylases and methyltransferases, also use an LXCXE motif to interact with pRB to participate in cell cycle regulation [7]. We have previously mutagenized the pRB pocket domain to map protein interaction sites and these results have been published [8-10]. This work revealed that discrete mutations in *RB1*, that only affect LXCXE interactions and not E2Fs, are limited to the coding region of exons 21 and 22 (Fig. 1). This is likely because the amino acids encoded by these exons are in close proximity in the three dimensional structure of the pRB pocket domain [11]. Others have also reported that the C-terminal region of pRB, that is phosphorylated by Cyclin/CDKs, can interact with and regulate access to the LXCXE binding cleft [12]. This region is encoded by exon 23. Based on these data we hypothesized that cancer derived mutant alleles of human *RB1 *in these three exons have the potential to specifically disrupt LXCXE-type interactions in isolation from other interactions with pRB's pocket domain. Our work demonstrates the existence of this class of mutation in human cancer. We also provide evidence that this type of mutation can compromise pRB's ability to regulate proliferation even when heterozygous with a wild type copy of the retinoblastoma gene.

## Results and Discussion

**Figure 2:**
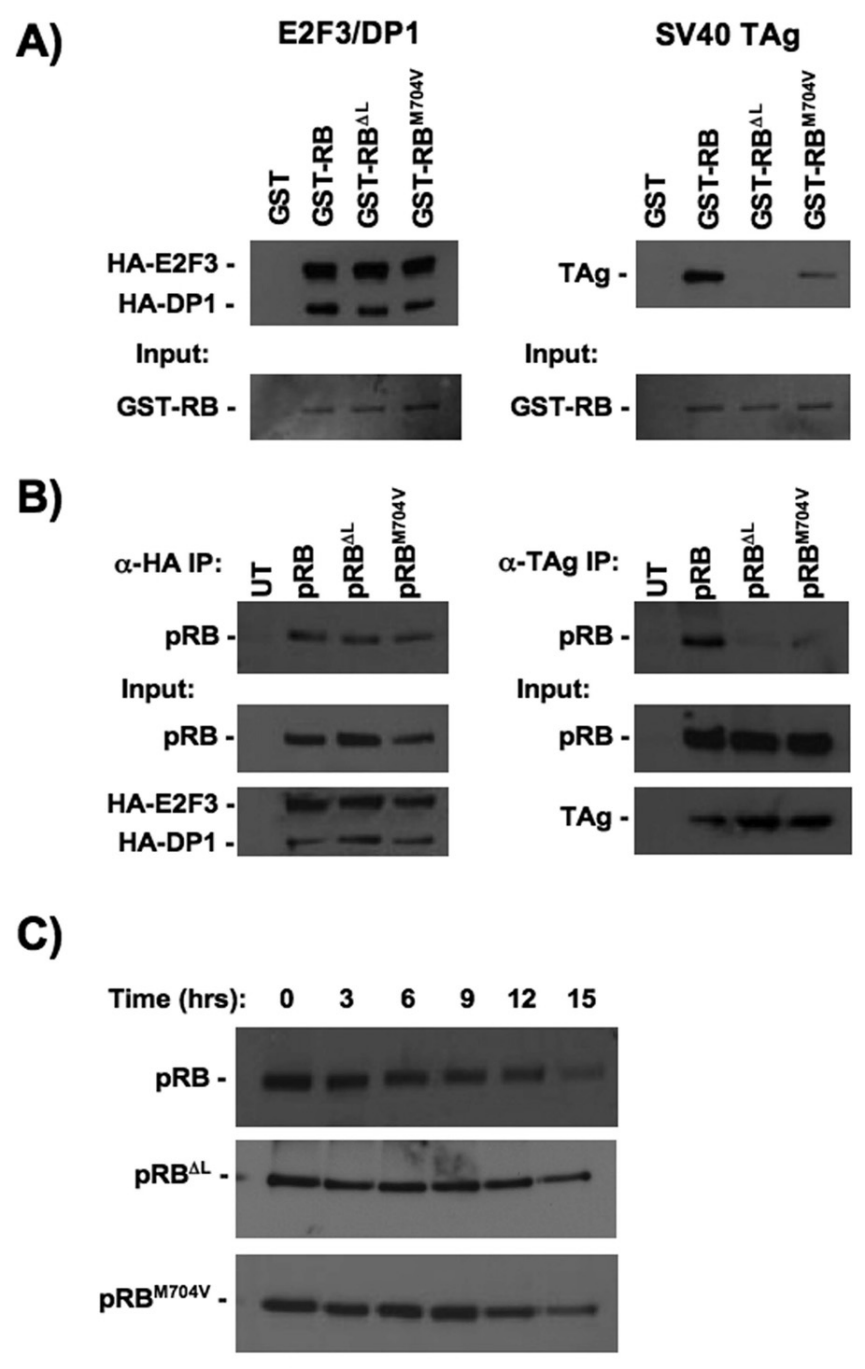
**The pRB^M704V ^protein is defective for LXCXE-dependent interactions**. (A) The ability of wild type or mutant forms of GST-RB to interact with TAg or E2F3/DP1 was determined by pull-down experiments. The input level of GST-RB proteins was determined by coomassie staining of SDS-PAGE gels, and the quantity of TAg or HA-E2F3/HA-DP1 that was precipitated with GST-RB was detected by western blotting. (B) Interactions were also assessed by co-immunoprecipitation of pRB with TAg or HA-E2F3/HA-DP1 proteins. In each case, the quantity of pRB present in precipitated complexes was determined by western blotting. UT indicates extracts that were generated from untransfected cells. (C) *RB1 *deficient cells transfected with wild type or mutant pRB were treated with cycloheximide, harvested at the indicated time points, and the quantity of pRB present was determined by western blotting.

To determine if pRB-LXCXE disrupting mutations arise in human cancer we initially searched the Retinoblastoma Genetics Database [13] and the Catalogue of Somatic Mutations in Cancer (COSMIC) database [14]. A number of missense mutations have been identified that are found in exons 21, 22 or 23 and we have generated expression constructs for them to enable further study (Fig. 1). Additional mutations in these exons exist beyond what we report here. However, they have either been previously characterized, appear to be redundant with mutations that we are studying, or are alleles that are very similar to those tested in our previous alanine scanning experiments. For example, the C706F variant found in small cell lung cancer [15] has been well-characterized in previous studies, and while it is unable to interact with LXCXE proteins, it is also incapable of interacting with E2F transcription factors, among other defects [16-19]. Furthermore, we expect that it is very similar to the retinoblastoma derived C706Y mutant. Based on this reasoning, the mutations listed in Fig. 1 were selected for use in this study because they are unique and uncharacterized. Mutant proteins containing these substitutions were tested for their ability to bind to LXCXE proteins, such as TAg, as a marker for LXCXE-dependent interactions (data not shown). Of the mutants tested, only M704V showed properties similar to the pRB^ΔL ^mutant protein that we generated based on crystallographic data to specifically disrupt this interaction (the ΔL mutant contains I753A, N757A, and M761A substitutions). The properties of the M704V protein are revealed in experiments using both GST-pulldown and immunoprecipitation approaches (Fig. 2A &2B). Like pRB^ΔL^, pRB^M704V^ retains the ability to interact with E2F3/DP1, but its ability to interact with SV40-TAg is greatly compromised. The similarity in specificity between our synthetic mutant pRB^ΔL ^and the cancer derived M704V variant is likely explained by the close proximity of the substituted amino acids in the crystal structure of the pRB pocket [11]. In addition to alterations in structure, mutations can also cause proteins to lose function due to a decrease in protein stability. To examine this possibility, cells expressing wild-type pRB, pRB^ΔL ^or pRB^M704V ^were treated with cycloheximide to block *de novo *protein synthesis. Cells were harvested every 3 hours after treatment for 15 hours and probed for pRB to monitor rates of protein degradation (Fig. 2C). The RB^M704V ^protein showed similar stability to wild type pRB and pRB^ΔL^, with a half-life ranging from 10 to 12 hours across a number of experiments, which is comparable to previous publications [20,21]. Taken together, our data suggests that the pRB^M704V ^mutant has a specific defect for LXCXE interactions and is stably expressed.

**Figure 3:**
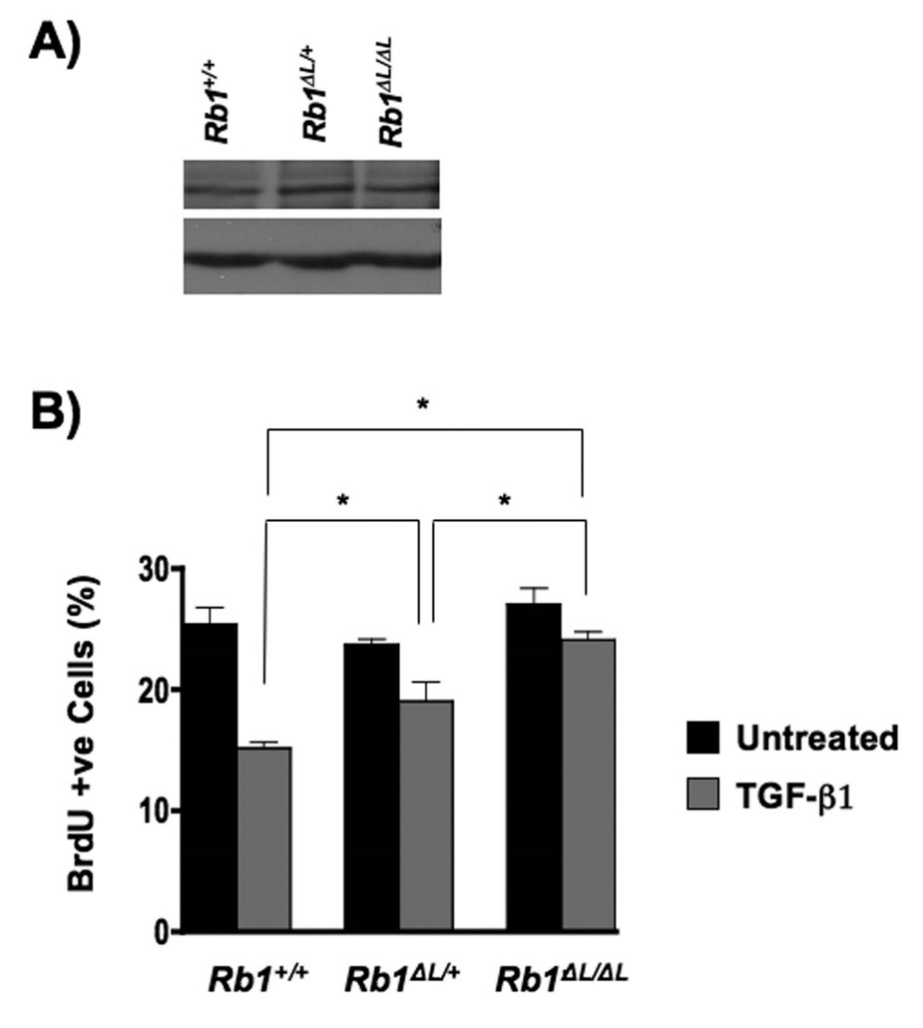
**Defective TGF-β growth arrest in *Rb1*^Δ*L/*^⁺ fibroblasts**. (A) The level of pRB expression was measured in *Rb1*^⁺*/*⁺^, *Rb1*^Δ*L/*⁺^ and *Rb1*^Δ*L/ΔL *^fibroblasts by western blotting. The upper blot shows pRB expression in these extracts and the lower blot shows Actin as a loading control. (B) All cells were treated with TGF-β for 24 hours, pulse labelled with BrdU for 1.5 hours and analyzed for incorporation by flow cytometry to determine the percentage of positive cells. The mean percentage of BrdU positive cells was determined from three independently treated cultures of cells. Means were compared between genotypes using a *t*-test and were found to be statistically significant (* *P *< 0.05).

In the initial report of the M704V variant of pRB, it was described as heterozygous [22]. Given that the retinoblastoma gene is thought to be functional until loss of heterozygosity removes the remaining wild type allele, we wondered how much the M704V mutant contributed to the pathogenesis of the tumor where it was found. To address the effects of heterozygosity of the M704V allele, we took advantage of a gene targeted, knock in mouse strain that possesses an analogous allele in its mouse *RB1 *gene (called *Rb1^ΔL^*). There are several advantages to using a targeted knock in model over cell culture systems. Initial characterization of the pRB^ΔL ^mutant revealed surprisingly few effects in cell culture assays; the mutant protein maintained its ability to induce a cell cycle arrest in *RB1 *deficient Saos2 cells as well as its ability to repress E2F target genes [8]. This was unexpected because the LXCXE binding cleft region of pRB is highly conserved and confers susceptibility to viral pathogens, suggesting that it must have an important function. To circumvent the limitations of cell culture systems, further studies were performed in fibroblast cells from *Rb1^ΔL  ^*mice. Using these cells, discrete defects in pRB^ΔL^'s ability to control the cell cycle were identified. More specifically, disruption of LXCXE interactions *in vivo *abrogates G1 arrest in response to exogenous stimuli such as DNA damage or transforming growth factor-β(TGF-β), but does not speed up G1-S phase progression in proliferating cultures of cells [23-25]. For these reasons, we tested the response of *Rb1*^⁺*/*⁺^, *Rb1*^*ΔL/*⁺^ and *Rb1*^*ΔL/ΔL  *^primary embryonic fibroblasts to TGF-β treatment. In this way we could carefully examine gene-dosage effects of the *Rb1*^*ΔL*^mutant on growth arrest in an otherwise normal cell. First, we tested the stability of the *Rb1*^*ΔL*^ allele *in vivo*, and found that the steady state levels of pRB in both *Rb1*^*ΔL/⁺*^ and *Rb1*^*ΔL/ΔL *^cells were similar to wild-type, confirming that this mutation does not affect protein stability (Fig. 3A). Cells were then treated with TGF-β for 24 hours, pulse labelled with bromodeoxyuridine (BrdU), and the percentage of BrdU positive cells was quantified by flow cytometry (Fig. 3B). While the *Rb1*^⁺*/*⁺^   fibroblasts exhibited a marked decrease in proliferation in response to TGF-β treatment, both the *Rb1*^*ΔL/* ⁺^ and the *Rb1*^*ΔL/ΔL *^cells displayed resistance to TGF-β growth arrest. Pair wise comparisons of the percentage of BrdU positive cells following TGF-β treatment between each genotype indicates that they are significantly different (*t*-test, *P *< 0.05). This reveals that loss of LXCXE interactions by even a single *Rb1 *allele significantly reduces TGF-β growth inhibition. The ability of the *Rb1*^*ΔL*^ mutation to confer resistance to TGF-β 's cytostatic effect is highly suggestive that the M704V variant of pRB can contribute to cancer susceptibility because even when heterozygous, mutations that disrupt LXCXE interactions compromise pRB's ability to regulate proliferation.

**Figure 4:**
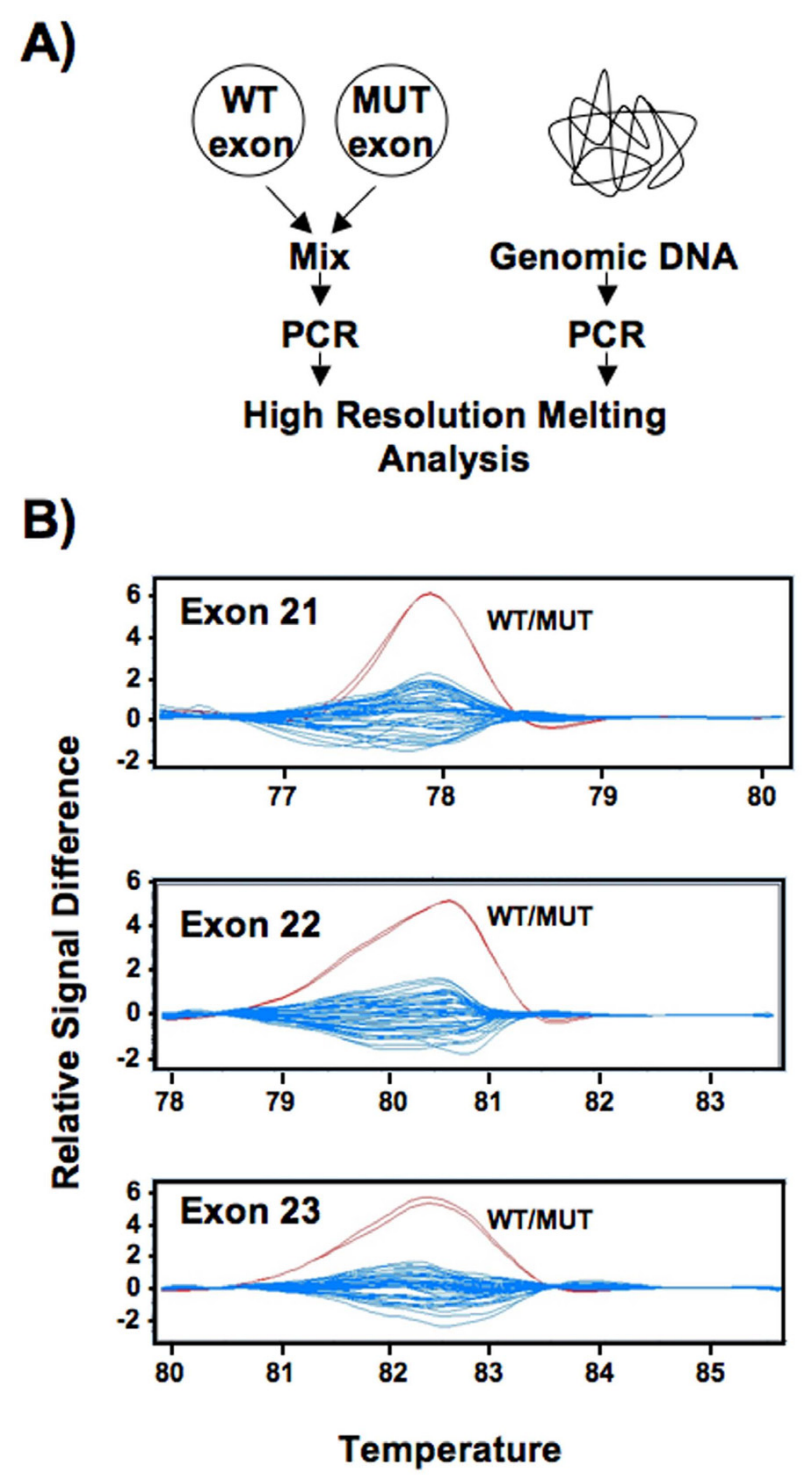
**High resolution melting analysis of *RB1 *exons 21, 22 and 23**. (A) To detect heterozygous sequence changes in *RB1*, constructs were created that encode either exon 21, 22 or 23 with or without single base pair substitutions. Equal quantities of these constructs were mixed, PCR amplified, and subjected to melting analysis alongside human genomic DNA samples. (B) Each DNA sample was analyzed in duplicate and the differences in melting profiles are plotted as a normalized difference plot. Representative difference plots for each exon are shown. In each case the pair of heterozygous plasmid controls (shown in red) is readily distinguishable from the homozygous human samples (shown in blue) by the Gene Scanning software as it assigns the colours.

The mammary specific growth control defects in *Rb1*^Δ*L*/*ΔL *^mice [24], along with the origin of the M704V variant, suggest that ΔL-like mutations may be found in breast and ovarian cancers. The connection between breast and ovarian cancer is well known and the requirement for LXCXE interactions in TGF-β growth arrest further suggests a link between this type of mutation and these cancers. Indeed, two recent studies suggest that as yet unidentified allelic variants of *RB1 *may modify the risk of breast and ovarian cancer [26,27]. For these reasons, we sought to identify more alleles of this type from breast and ovarian cancer patients. To search for these alleles, we devised a high resolution melting (HRM) assay. HRM is a highly sensitive method that uses the distinct melting curves of each region of DNA to identify sequences that differ from a known standard. Again, the target of this analysis was *RB1 *exons 21, 22, and 23 because they can be altered to create alleles like M704V. To test the sensitivity of HRM at this locus, primers were designed to amplify each exon individually and plasmid constructs were generated that encode either exon 21, 22 or 23 with or without single base pair substitutions from previously characterized *RB1 *mutations. To detect heterozygous sequence changes in *RB1*, equal quantities of the wild type and mutant version of each construct were mixed, PCR amplified, and subjected to melting analysis alongside human genomic DNA samples (Fig. 4A). When the melt profile is compared to wild type control DNA, a shift in the melting curve is observed. When analyzed by Gene Scanning software, the control heterozygous samples were readily distinguished in a difference plot, validating the sensitivity of this assay for each exon (Fig. 4B). In total 627, genomic DNA samples were analyzed, representing individuals classified as high risk for inherited breast and/or ovarian cancer. To search for mutations in primary tumor specimens, 50 breast and 21 ovarian tumor samples were obtained. These were PCR amplified and directly sequenced using primers specific to each exon because they may be homozygous at the *RB1 *locus and thus indistinguishable from wild type in our HRM analysis. The results from both methods of screening are summarized in Table 1. No new alleles were detected by this approach.

**Table 1:** Summary of mutation detection.

	Blood samples		Tumor samples			
			**Breast**		**Ovarian**	

	**Screened**	**Mutations**	**Screened**	**Mutations**	**Screened**	**Mutations**

Exon 21	627	0	50	0	21	0
Exon 22	627	0	50	0	21	0
Exon 23	627	0	50	0	21	0

To detect heterozygous sequence changes, peripheral blood samples from breast and/or ovarian cancer patients were subjected to HRM analysis using primers specific to exons 21, 22 and 23. To detect potentially homozygous sequence changes from breast and ovarian tumor samples, PCR amplicons of these exons were directly sequenced.

## Conclusions

In this study we sought to identify cancer-associated mutations that specifically target the region of *RB1 *encoding the LXCXE binding cleft. In this way, our goal was to search for distinct *RB1 *alleles that are compromised for just one aspect of its function. We characterized an ovarian cancer derived variant of *RB1 *that possesses biochemical properties that are comparable to our previously published *Rb1*^*ΔL *^mutant allele. We also showed that a single mutant allele of *Rb1 *can significantly decrease the response to TGF-β mediated growth arrest, indicating that the retinoblastoma gene displays haploinsufficiency for growth control. While we were unable to find more examples of this type of allele, our work suggests that cancer types that are characterized by resistance to TGF-β growth arrest and maintain heterozygosity for *RB1 *may possess this type of mutant allele as a means of overcoming growth inhibitory mechanisms that limit tumor formation.

## Methods

### Protein interaction analysis

Glutathione S-transferase (GST) pull-down experiments were performed as described in Dick et al. [8]. The GST-RB constructs contain the large pocket of pRB, encompassing amino acids 379 to 928. The GST-RB^ΔL ^and GST-RB^M704V ^contain I753A, N757A and M761A or M704V substitutions, respectively. In each case, recombinant proteins (or GST as a control) were incubated with whole-cell extracts from C33A cells transfected with either CMV-HA-E2F3 and CMV-HA-DP1, or CMV-TAg. Bound proteins were detected by western blotting using 12CA5 hybridoma supernatant for HA-tagged E2F3/DP1, or mouse monoclonal antibody PAb419 (CalBiochem) for SV40-TAg, followed by a peroxidase conjugated anti-mouse IgG secondary antibody. Input levels of GST-fusions were detected by Coomassie staining.

Immunoprecipitations were performed essentially as described in Siefried et al. [28]. The pRB constructs tested contain either the full-length wild type *RB1 *cDNA (CMV-RB) or *RB1 *cDNA that has been mutated to create the desired amino acid substitutions (CMV-RB^ΔL ^and CMV-RB^M704V^). In brief, C33A cells were transfected with 10 μg of each CMV-RB construct in addition to either 5 μg of CMV-TAg or 5 μg each of CMV-HA-E2F3 and CMV-HA-DP1. To precipitate TAg immune complexes, the PAb419 antibody (CalBiochem) was used, whereas 12CA5 hybridoma supernatant was used to pull down HA-containing immune complexes. In each case, bound pRB was detected by western blotting using the G3-245 α-pRB antibody (BD Pharmingen), as above. HA-E2F3, HA-DP1 and TAg input levels were also detected by western blotting.

### Protein stability assay

To assess protein stability, 2.6 × 10^6 ^C33A cells were transfected by Ca_2_PO_4 _with 45 μg of CMV-RB, CMV-RB^ΔL ^or CMV-RB^M704V ^in a 15 cm plate. Twenty-four hours post-transfection cells were replated into 6 replicate 6 cm plates. Twenty-four hours after replating, cells were treated with 100 μg/mL cycloheximide and harvested after 0, 3, 6, 9, 12 and 15 hours. To detect pRB, 10 μg of total protein was analyzed by western blotting as described above. Protein levels were quantified by densitometry using a Bio Rad Gel Doc XR gene imager and half-lives were calculated accordingly. To assess the expression of pRB *in vivo*, asynchronously proliferating *Rb1* ⁺^*/*^⁺, *Rb1*^*ΔL/*^⁺ and *Rb1*^*ΔL/ΔL*^ MEFs were harvested and 50 μg of total protein was analyzed by western blotting using the *Rb1 *4.1 hybridoma, followed by a peroxidase conjugated anti-mouse IgG secondary antibody. The hybridoma developed by Julien Sage was obtained from the Developmental Studies Hybridoma Bank developed under the auspices of the NICHD and maintained by The University of Iowa, Department of Biology, Iowa City, IA 52242.

### TGF-β growth arrest assays

Asynchronously proliferating *Rb1*^⁺*/*⁺^, *Rb1*^*ΔL/*⁺^ and *Rb1*^*ΔL/ΔL*^ MEFs were treated with 100 pM TGF-β1 (R&D systems) for 24 hours. Cells were then pulse labelled with BrdU (RPN201V1, Amersham Biosciences) for 1.5 hours. BrdU incorporation was quantified using flow cytometry on a Beckman-Coulter EPICS XL-MCL instrument, as previously described [29].

### Patient samples

Peripheral blood DNA samples (627 specimens) from human breast and/or ovarian cancer patients were obtained from the London Health Sciences Centre, Molecular Diagnostics Laboratory (London, ON). All samples were post-testing material from individuals that meet Ontario provincial referral criteria because they are under the age of 35 (173), have three or more cases of breast or ovarian cancer on the same side of the family (155) or otherwise have a pedigree that is strongly suggestive of hereditary breast/ovarian cancer. Frozen breast tumor samples were obtained from the Ontario Tumour Bank. Samples were chosen at random (not based on histological characteristics) from patients between 38 and 87 years of age. All tumor material was derived from the primary site. DNA was isolated from these samples using standard techniques. DNA from ovarian tumor samples was prepared from passage two cells that were isolated from patient ascites. Ovarian samples were from patients between 25 and 85 years of age. DNA was provided courtesy of the Translational Ovarian Cancer Program at the London Health Sciences Centre.

### High resolution melting (HRM) analysis

HRM analysis was conducted using the Roche Lightcycler 480 HRM kit. In brief, each 20 μL reaction consisted of 10 μL of 2× Master Mix, 0.1875 μM of each primer, 3.0 to 4.0 mM of MgCl_2 _and 50 ng of genomic DNA. As a positive control, test constructs encoding individual exons with either a single nucleotide change (mutant) or without (wild type) were mixed in equal quantities and 0.1 pg of the resulting mixture was used in place of a genomic DNA sample. The mutant constructs for exon 21 and exon 22 each contain a single G to T substitution (g.160839G>T and g.162027G>T) from previously reported cancer-causing *RB1 *alleles [30], whereas the construct for exon 23 contained a previously reported C to G substitution (g.162241G>C)[31]. To amplify each exon the following primer pairs were used: exon 21 cagtatggaaagaaataactctgtag and gtgaatttacataataaggtcagacag, exon 22 gcccccgccgttactgttcttcctcagacattcaa and cccccgcccgaatgttttggtggacccatt and exon 23 gcggcccgccgcccccgccgcttccaccagggtaggtcaa and gccgggcgcgcccccgcccgggatcaaaataatccccctctcat. The amplification and melt analysis were conducted sequentially in the Roche Lightcycler 480. First, samples were incubated at 95°C for 10 minutes, followed by 50 cycles of: 95°C for 10 seconds, a touch down of 65 to 55°C (exon 21) or 70 to 65°C (exons 22 and 23) for 20 seconds, 72°C for 10 seconds. The samples were then heated to 95°C to generate a melt curve. All samples were run in duplicate and each plate contained duplicate positive controls. Lightcycler 480 Gene Scanning software was used to normalize the data and generate difference plots.

### Sequencing

To detect sequence changes in tumor DNA samples, PCR products were generated and directly sequenced (McGill University and Genome Quebec Innovation Centre). Exon 21 products were obtained and sequenced using primers 21F (ttgggttaaacacttcatgtagac) and 21R (cctatgttatgttatggatatggatt). Exons 22 and 23 were amplified in a single reaction using primers 22F (tataatatgtgcttcttaccagtcaa) and 23R (aagcaaatatgagtttcaagagtctagc) and sequenced using primers 22F and 23R2 (gcgttgcttaagtcgtaaatagatt).

## Abbreviations

pRB: retinoblastoma protein; *RB1*: human retinoblastoma gene; *Rb1*: murine retinoblastoma gene; TGF-β : transforming growth factor-beta; BrdU: bromodeoxyuridine; HRM: high resolution melt.

## Competing interests

The authors declare that they have no competing interests.

## Authors' contributions

SAH planned and performed the experiments in Fig. 2 and 4, and Table 1. SMF planned and performed the experiments in Fig. 3. JD initially demonstrated the properties of the M704V mutant. PA planned and supervised the mutation detection experiments in Fig. 4 and Table 1. FAD and SAH wrote the manuscript. All authors have approved the content of this manuscript.
